# Menthol increases human glioblastoma intracellular Ca^2+^, BK channel activity and cell migration

**DOI:** 10.1186/1423-0127-16-90

**Published:** 2009-09-24

**Authors:** Robert Wondergem, Jeremy W Bartley

**Affiliations:** 1 Department of Physiology, James H Quillen College of Medicine, East Tennessee State University, PO Box 70,576, Johnson City, Tennessee 37614-1708, USA

## Abstract

This study examined the effect of menthol, an agonist for transient receptor potential melastatin 8 (TRPM8) ion channels, to increase intracellular Ca^2+ ^concentration, [Ca^2+^]_i_, in human glioblastoma cells (DBTRG cells), which resulted in activation of the large-conductance Ca^2+^-activated K^+ ^membrane ion channels (BK channels). Voltage ramps applied over 300 ms from -100 to 100 mV resulted in membrane currents with marked inwardly- and outwardly-rectifying components. Paxilline (2 μM) abolished the outwardly-rectifying current. Outwardly-rectifying on-cell patch currents were increased markedly by menthol (100 μM) added to the bath. The estimated on-cell conductance of these channels was 253 pS. Kinetic analysis showed that added menthol increased channel open probability and mean open frequency after 5 min. In a similar time course menthol increased [Ca^2+^]_i_, and this increase was abolished either by added paxilline, tetraethylammonium ion or by Ca^2+^-free external solution. Finally, menthol stimulated the rate of DBTRG cell migration into scratch wounds made in confluent cells, and this also was inhibited by paxilline or by tetraethylammonium ion. We conclude that menthol, a TRPM8 agonist, increases DBTRG cell [Ca^2+^]_i _that in turn activates membrane BK ion channels. Inhibition of BK channels by paxilline reverses menthol-stimulated increase of [Ca^2+^]_i _and of cell migration. Thus, BK channels function to maintain elevations in [Ca^2+^]_i _needed to sustain increases in DBTRG cell migration.

## Background

Glioblastoma multiforme (GBM) has a particularly grim prognosis with a median survival time of 15 months [1]. The extreme invasive property of this tumor, along with propensity for phenotypic changes to occur within the population of invading cells compared with the primary tumor mass, present unyielding dilemmas for effective surgical, chemo- or radiation therapy. Thus, efforts to understand the mechanisms by which tumor cells migrate and invade will provide useful information to alleviate the progression of this disease.

We have shown recently that a transient receptor potential melastatin 8 ion channel, TRPM8, is expressed in a human glioblastoma cell line (DBTRG), and its function contributes to increasing the intracellular Ca^2+ ^concentration, [Ca^2+^]_i_, that is necessary for cell migration and, presumably, tumor invasion [2]. TRPM8 is highly expressed in prostate and other cancer cells [3], but, apart from its principal function in neurons as a detector of environmental cold [4], its physiological and pathological function in epithelia and cancer cells is unknown [5].

Others have shown that the large-conductance Ca^2+^-activated K^+ ^ion channels (ie. maxi-K or BK) are over-expressed in human glioma cells [6]. These BK channels are the product of spliced-variant *hslo *gene and have enhanced sensitivity to [Ca^2+^]_i _[7,8]. They function to enhance the migration and invasive property of gliomas presumably by contributing to the plasma membrane ionic fluxes that underscore cell volume regulation, particularly as these cells alter their volume through the restricted intercellular spaces available to tumor cells invading the brain parenchyma [9].

In this study we report the integrative function of BK ion channels in relation to the increase in cell migration by menthol, the putative agonist of TRPM8 ion channels. We show that menthol increases [Ca^2+^]_i _necessary for stimulating glioblastoma cell migration, and that blocking BK channels abolishes the menthol-stimulated increase in [Ca^2+^]_i _as well as cell migration.

## Materials and methods

### Cell culture and materials

Human GBM cells (DBTRG cells) were a gift from G.F. Vande Woude, Van Andel Research Institute, Grand Rapids, MI. Cells were grown in Dulbecco's MEM supplemented with 10% FBS (Invitrogen) and 100 IU-100 μg/ml penicillin-streptomycin (Sigma).

### Whole-cell Voltage Clamp and On-cell Patch Clamp Technique

DBTRG cells were superfused on a microscope stage at room temperature with a standard external salt solution containing (in mM): 150 NaCl, 6 KCl, 1 MgCl_2_, 1.5 CaCl_2_, 10 HEPES [*N*-(2-Hydroxethyl)piperazine-*N'*-(2-ethanesulfonic acid)], 10 glucose, and pH 7.4 (1N HCl). In some instances KCl was increased from 6 mM to 60 mM by isosmotic substitution for NaCl. Whole-cell voltage clamp pipettes were fabricated by means of a Brown/Flaming micropipette puller (Sutter Instr. Co, Novato, CA) and were filled with (in mM): 140 KCl, 6 CaCl_2 _(302-nM free Ca^2+^; computed by WCaBuf software from guy.droogmans@med.kuleuven.ac.be), 2 MgCl_2_, 11 EGTA, 50 HEPES, and pH 7.2 (1N KOH). The pipettes were 4-5 MΩ in the bath solution, and whole-cell voltage-clamp measurements were performed by standard technique [10]. On-cell patch clamp pipettes were filled with (in mM): 20 CsCl, 100 aspartic acid, 0.1 CaCl_2_, 1 MgCl_2_, 5 EGTA 10 HEPES, and pH 7.4 (1N CsOH). (1-2 MΩ in bath solution).

### Ca^2+ ^Measurements by fluorescence imaging of Fura2

Cells on glass coverslips were loaded with Fura2 and [Ca^2+^]_i _was measured by inverted microscopy (AccuScope 3030) [11]. Filter-wheel and data acquisition were controlled by the InCyte2 software (Intracellular Imaging, Inc, Cincinnati). [Ca^2+^] was determined by interpolation from a standard curve generated from a Ca^2+ ^calibration buffer kit #2 (Molecular Probes) and Fura2/K_5_-salt.

### Cell migration assay

DBTRG cells were plated into 12-well tissue culture plates and grown until confluent. A disposable pipette tip (250 μL) was used to scratch a wound on midline of the culture well. Photomicrographs were taken at various times, and wound width was measured and recorded using MetaMorph software (Molecular Devices), which was calibrated at 1.04 μm/pixel by the grid on a hemocytometer.

### Data analysis

Results are expressed as mean ± SEM. Differences among means were determined by Student's paired *t*-test, p < 0.05. Electrophysiological data were analyzed using WinASCD software (Guy Droogmans, Katholieke Universiteit, Leuven, Belgium, ftp://ftp.cc.kuleuven.ac.be/pub/droogmans/winascd.zip) Migration rates were determined by regression analysis of wound width versus time (hrs), and treatments were deemed effective compared with control if the null-hypothesis of common slopes (*H*_o_: b_1_-b_2 _= 0) was rejected at p < 0.05.

## Results

**Figure 1:**
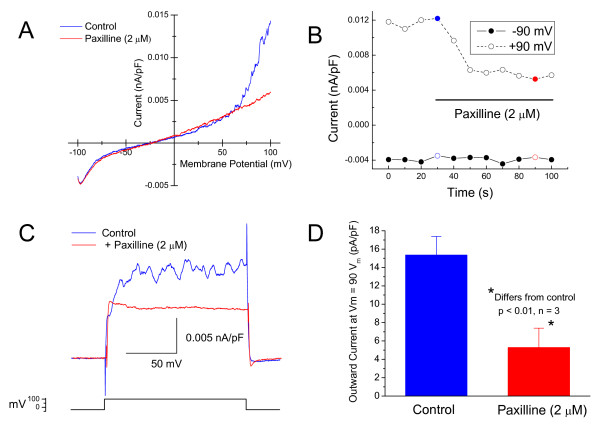
**Whole-cell voltage clamp measurements of human glioblastoma cells**. Holding potential = -30 mV. ***A***. Current-voltage plots obtained during voltage ramps from -100 to 100 mV (300 ms duration). Plots were obtained before (control) and after added paxilline (2 μM). ***B***. Whole-cell currents obtained at -90 and 90 mV plotted versus time before and after added paxilline (2 μM). Reversed symbols are from I-V plots shown in A. ***C***. Whole-cell currents recorded during voltage steps from -30 to 90 mV before and after added paxilline (2 μM). ***D***. Summary results (as in C) of the effects of paxilline on outward, whole-cell currents recorded at voltage steps to 90 mV.

DBTRG whole-cell membrane currents were recorded during voltage ramps (300 ms) from -100 to 100 mV (HP = -30 mV), Figure 1. The control I-V plot displayed both inwardly and outwardly rectifying currents; however, added paxilline (2 μM) quickly reduced the outwardly rectifying current, Figure 1A. The time course for this inhibition by paxilline was rapid as is evident from the plot of whole-cell current at -90 and 90 mV versus time, Figure 1B. Paxilline had similar effects on outward currents generated by voltage steps from -30 mV (holding potential) to 90 mV, Figure 1C, and this inhibition was significant, Figure 1D.

**Figure 2:**
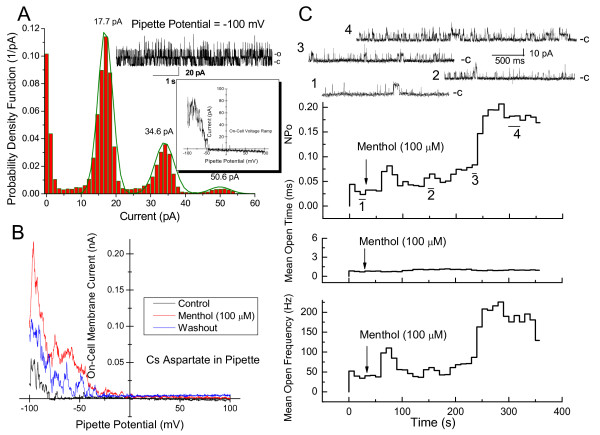
**On-cell patch clamp measurements of human glioblastoma cells**. ***A***. Relative frequency of channel opening (probability density function) plotted versus channel current. Peaks at specified currents are indicated by Gaussian fit. The analysis was of the displayed readout of patch current obtained from 6.3 s of on cell recording at pipette potential = -100 mV; (c)-closed state; (o)-open state. (*inset*) Membrane/patch current plotted as a function of patch pipette potential from -100 to 100 mV (300 ms duration). ***B***. On-cell outward patch currents obtained from voltage ramps (-100 to 100 mV; 300 ms duration) before, during and after menthol treatment of the cell. ***C***. Kinetic analysis of on-cell channel recording (-100 mV pipette potential) before and after menthol added to the bath (100 μM). Numbered traces correspond to numbered segments of the analysis.

Paxilline is known to inhibit the large-conductance Ca^2+^-activated K^+ ^channels (BK) [12,13]. As a result, we performed on-cell patch-clamp recordings of DBTRG cells to determine if the outwardly rectifying currents might be attributable to BK ion channels. The inset in Figure 2A shows the current-voltage (I/V) plot obtained from an on-cell voltage ramp (-100 to 100 mV pipette potential; 300 ms duration) applied to a DBTRG cell. Outward currents were clearly evident in the pipette potential range of -100 to -45 mV. Readout of channel currents recorded on-cell at a pipette potential of -100 mV is shown above the inset, Figure 2A. A histogram of relative frequency versus these channel currents shows three distinct peaks, with the smaller peaks at 34.6 and 50.6 pA, respectively, approximating multiples of the unit current of 17.7 pA. Assuming the membrane potential of the DBTRG cells at -30 mV, these currents yield a channel unitary conductance of approximately 253 pS. This magnitude agrees well with the range of conductance reported for BK channels [7,14].

Since added menthol increases [Ca^2+^]_i _and increases the rate of migration of DBTRG cells [2], we performed measurements to determine whether added menthol correspondingly increases BK channel activity. On-cell membrane currents in response to voltage ramps from -100 to 100 mV were recorded from a DBTRG cell, Figure 2B. Outward currents were evident at very positive transmembrane voltages. The magnitude of this current and the range of transmembrane voltage in which they occurred increased markedly within five minutes of added menthol (100 μM). This reversed following washout of menthol. Similar effects were evident from kinetic analysis of single channel activity recorded during on-cell recording from a DBTRG cell (-100 mV) pipette potential, Figure 2C (bottom). Channel open probability (NPo), mean open time (ms) and mean open frequency (Hz) were computed from read-out of continuous on-cell recording before and after added menthol (100 μM). Segments of channel read-outs corresponding to the numbers shown in the channel open-probability plot are also shown, Figure 2C (top). Added menthol increased DBTRG on-cell BK ion channel open probability and mean open frequency within 4-5 min. However, menthol had no affect on mean open time.

**Figure 3:**
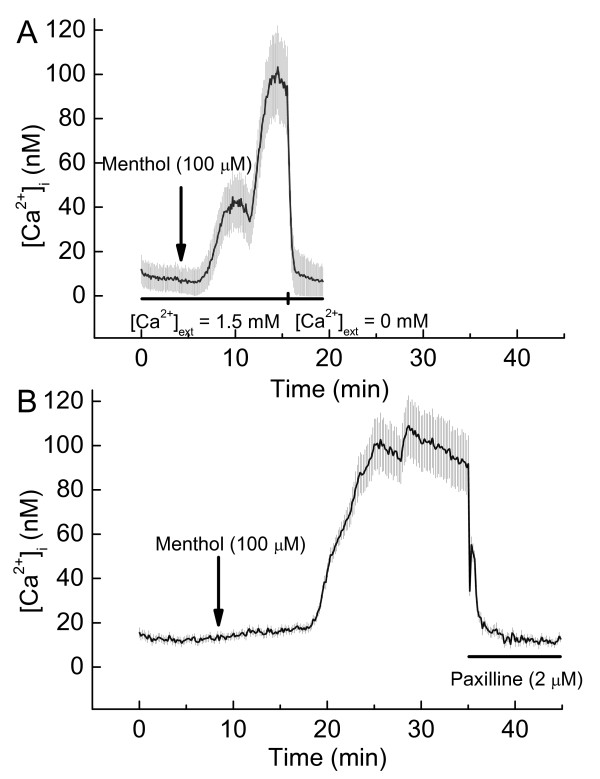
**Effect of added menthol on intracellular [Ca^2+^], [Ca^2+^]_i_, of human glioblastoma cells**. ***A***. Effect of switching from Ca^2+^-containing external solution to Ca^2+^-free external solution on menthol-stimulated increases of [Ca^2+^]_i_; Mean ± SE, n = 21 cells. ***B***. Effect of added paxilline (2 μM) on menthol-stimulated increases of [Ca^2+^]_i_; Mean ± SE, n = 26 cells.

**Figure 4:**
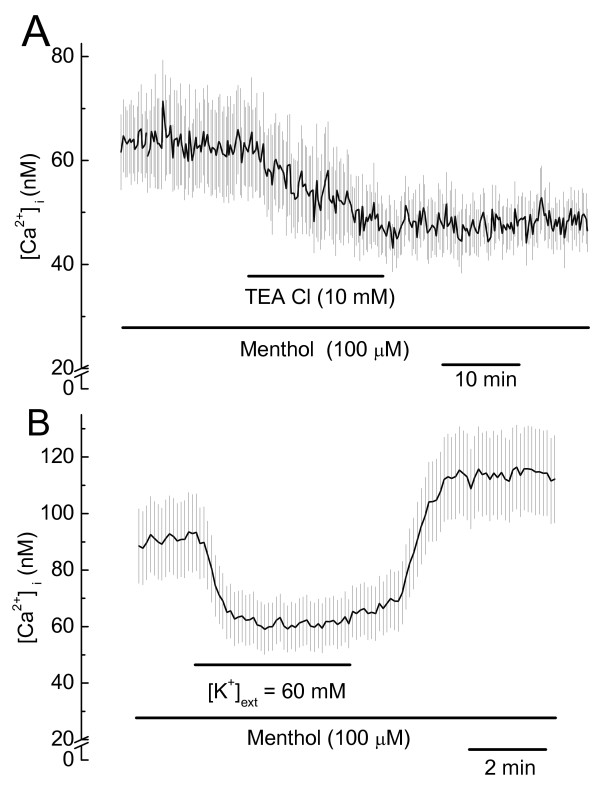
**Effect of tetraethylammonium chloride (TEA Cl) and membrane depolarization on menthol-stimulated increases of [Ca^2+^]_i_, in human glioblastoma cells**. ***A***. Effect of TEA (10 mM) on menthol-stimulated increases in [Ca^2+^]_i_; Mean ± SE, n = 17 cells. ***B***. Effect of membrane depolarization by means of a ten-fold increase in external K^+ ^concentration on menthol-stimulated increases of [Ca^2+^]_i _in human glioblastoma cells; Mean ± SE, n = 17 cells.

To test further whether this effect of added menthol on ion channel activity might be attributable to increased [Ca^2+^]_i _and, therefore, to BK channels, we measured effects of menthol on DBTRG [Ca^2+^]_i_. Menthol increased DBTRG [Ca^2+^]_i _within 5 to 10 min, Figure 3A and 3B. In spite of this delay in onset, the [Ca^2+^]_i _increased markedly and appeared in two waves. The increase in [Ca^2+^]_i _reversed rapidly and completely either by eliminating nominal Ca^2+ ^from the external solution, Figure 3A, or by adding paxilline to the medium superfusing the cell, Figure 3B. To rule out alternate effects of paxilline [15,16] we also measured the effects of tetraethylammonium ion (TEA) on DBTRG [Ca^2+^]_i _since it inhibits BK channels in human glioma cells [8]. TEA also reduced DBTRG [Ca^2+^]_i_, but the inhibition was slower and smaller than that of paxilline, Figure 4A.

It is likely that BK channel activity increases or sustains the transmembrane potential, which in turn maintains a favorable electrochemical potential for Ca^2+ ^influx into DBTRG cells. As an initial step to test this possibility we determined that the membrane depolarization from a ten-fold increase in external [K^+^] resulted in a reversible decrease in menthol-stimulated [Ca^2+^]_i_, Figure 4B.

**Figure 5:**
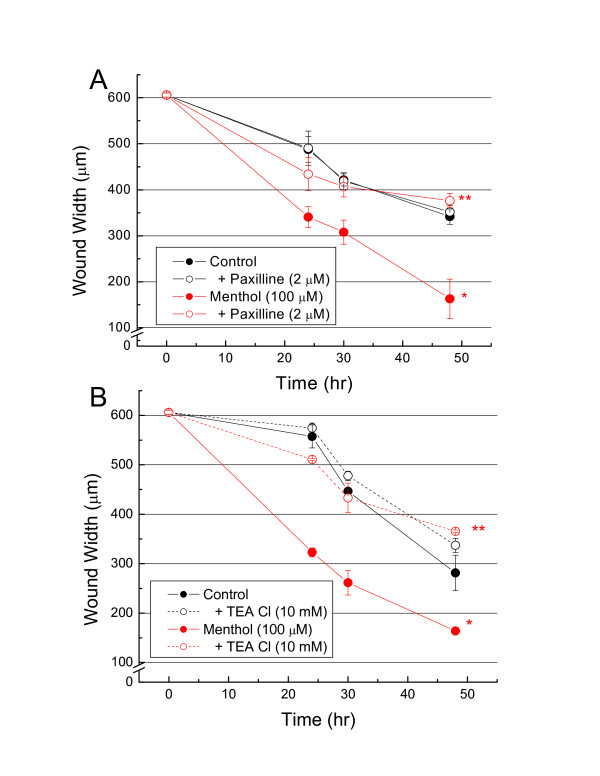
**Effect of menthol and paxilline or TEA, alone or in combination, on migration of human glioblastoma cells as determined by rate of closure of 'scratch-wounds' made in confluent cell cultures**. ***A***. Effect of paxilline (2 μM); ***B***. Effect of TEA (10 mM). *Differs from control, p < 0.001; **Differs from menthol, p < 0.001. Each point = Mean ± SE, n = 3.

In light of our observations that paxilline inhibit outwardly-rectifying membrane currents and that paxilline and TEA reverse menthol-stimulated increases in [Ca^2+^]_i _in DBTRG cells, we performed experiments to determine whether paxilline or TEA affect menthol-stimulated migration of DBTRG cells [2]. Menthol stimulated DBTRG cell migration into scratch wounds made on confluent cultures as shown previously [2], Figure 5. However, paxilline (2 μM) added simultaneously with menthol inhibited the latter's stimulation of migration, Figure 5A. In comparison, paxilline alone did not affect the rate of DBTRG cell migration, Figure 5A. Identical inhibition of menthol-stimulated migration occurred with added TEA, Figure 5B.

## Discussion

Calcium is an important intracellular signaling molecule that regulates many cellular functions including motility [17]. This pertains to tumor cells, because Ca^2+ ^pumps and channels serve as targets for therapeutic agents that limit tumor growth and metastasis [18]. We have shown previously that menthol stimulates influx of Ca^2+ ^into human glioblastoma cells, increases [Ca^2+^]_i _and, thus, increases the rate of cell migration [2]. The present findings expand these findings by showing that menthol-stimulated increases in [Ca^2+^]_i _activate membrane BK ion channels. The latter are widely expressed in human glioma cells [6], and they likely play a role regulating cell volume and, thus, cell motility [9].

The paxilline-sensitive outward current present in DBTRG cells implies they express BK ion channels. Their unitary conductance of 253 pS also agrees with that of BK channels [14], which are over expressed in human glioma cells [7] to an extent that functionally excludes other Ca^2+^-activated K^+ ^channels [19]. Menthol treatment increases BK channel activity, and the time course parallels menthol-stimulated increases in [Ca^2+^]_i_. The latter increases BK channel open probability and open frequency. Surprisingly, the rise in [Ca^2+^]_i _did not increase channel mean open time, which suggests an alternatively spliced isoform of the α subunit gene [20]. Indeed, such an isoform is expressed by various human gliomas [7].

These results also show that menthol stimulates influx of extracellular Ca^2+^. This is consistent with expression of TRPM8 ion channels by DBTRG cells [2]; however, it is not unequivocal that menthol stimulates TRPM8 channels in the plasma membrane. The menthol concentration to effect this rise in [Ca^2+^]_i _is relatively high, and the onset of this increase varies with time. The latter may result from autocrine/paracrine secretion of hepatocyte growth factor, whose concentration in the medium and ancillary effects on [Ca^2+^]_i _may depend on cell density [2,21]. It also could reflect time needed for insertion of TRPM8 ion channels into the plasma membrane [22], or it could reflect an intacellular site for TRPM8 ion channels. Bidaux et al reported expression of a TRPM8 truncated splice variant in the endoplasmic reticulum [23]. We reported expression of two TRPM8 proteins by DBTRG cells, one of the size predicted by the cDNA sequence and one truncated and found in plasma membrane and microsomal fractions [2]. Thus, menthol could effect increases in [Ca^2+^]_i _by its release from the endoplasmic reticulum and by its influx. So, it is noteworthy that menthol-stimulated rise in [Ca^2+^]_i _occurred in successive waves. Whether this involves store-operated Ca^2+ ^entry requires further investigation; however, it also is noteworthy that Orai1 and STIM1 are critical for breast tumor cell migration and metastasis [24].

The reversal of menthol-stimulated increases in [Ca^2+^]_i _by both added paxilline/TEA and Ca^2+^-free external solution indicates that BK channels play a role in maintaining elevated [Ca^2+^]_i_. Here the activation of BK channels may sustain or increase the magnitude of the transmembrane potential and, thus, maintain a favorable electrochemical potential for cation influx. Indeed, increasing external K^+ ^from 6 to 60 mM by isomolar substitution for Na^+ ^resulted in rapid diminution of [Ca^2+^]_i _that reversed on restoration of control solution.

The rise in [Ca^2+^]_i _is necessary for DBTRG cell migration, but the present study does not differentiate between global versus local increases in [Ca^2+^]_i_. It is becoming increasingly evident that Ca^2+^-activated K^+ ^channels are controlled by nano/micro domains of [Ca^2+^]_i _[25,26]. This may account for the discrepancy of the inhibitory effects of paxilline on BK channels and cell migration compared with another study showing that inhibition of BK channels increases the rate of migration of single human glioma cells in free range motion on a culture plate [27]. Scratch wounds result in a distinct polarity to migrating DBTRG cells, and blocking BK channels by paxilline inhibits their menthol-stimulated migration. Paxilline also blocks transwell migration of glioblastoma cells [19], which is an assay that subsumes polarity to cell migration. These latter assays likely replicate the polarity of cells found on the leading edge of an invasive glioblastoma. As such, Ca^2+^-activated K^+ ^channels may function to maintain local increases in [Ca^2+^]_i _necessary for molecular events of cell migration either at the membrane microdomains of the advancing lamellipodium [26] or those at the trailing footplate [28]. We cannot rule out alternate effects of paxilline; however, these occur at higher concentrations than used here to inhibit BK ion channels [15,16]. Moreover, TEA also inhibited menthol-stimulated increases in [Ca^2+^]_i _and cell migration.

## Conclusion

We have shown that menthol, a known agonist of TRPM8 ion channels found in DBTRG human glioblastoma cells, stimulates the migration of DBTRG human glioblastoma cells into scratch wounds and increases [Ca^2+^]_i _which, thereby, increases activity of BK ion channels. Inhibition of BK channels by paxilline or TEA abolishes the menthol-stimulated increases in [Ca^2+^]_i _and cell migration. We conclude that BK ion channels play an important role in cellular mechanisms that increase [Ca^2+^]_i _needed to increase the rate of cell motility and tumor invasion.

## Abbreviations

GBM: Glioblastoma multiforme; DBTRG cells: human glioblastoma cells; TRPM8: transient receptor potential melastatin 8 ion channel; BK: large-conductance Ca^2+^-activated K^+ ^ion channels; [Ca^2+^]_i_: intracellular Ca^2+ ^concentration; FBS: fetal bovine serum; HEPES: *N*-(2-Hydroxethyl)piperazine-*N'*-(2-ethanesulfonic acid); TEA Cl: Tetraethylmmonium chloride.

## Competing interests

The authors declare that they have no competing interests.

## Authors' contributions

RW conducted the electrophysiology, cell migration studies, and statistical analyses and drafted the manuscript. JWB carried out calcium measurements and helped draft the manuscript. Both authors read and approved the final manuscript.
